# Vitamin D receptor-binding site variants affect prostate cancer progression

**DOI:** 10.18632/oncotarget.18271

**Published:** 2017-05-26

**Authors:** Victor C. Lin, Shu-Pin Huang, Huei-Ju Ting, Wen-Lung Ma, Chia-Cheng Yu, Chao-Yuan Huang, Hsin-Ling Yin, Tsung-Yi Huang, Cheng-Hsueh Lee, Ta-Yuan Chang, Te-Ling Lu, Bo-Ying Bao

**Affiliations:** ^1^ Department of Urology, E-Da Hospital, Kaohsiung 824, Taiwan; ^2^ School of Medicine for International Students, I-Shou University, Kaohsiung 840, Taiwan; ^3^ Department of Urology, Kaohsiung Medical University Hospital, Kaohsiung 807, Taiwan; ^4^ Department of Urology, Faculty of Medicine, College of Medicine, Kaohsiung Medical University, Kaohsiung 807, Taiwan; ^5^ Graduate Institute of Medicine, College of Medicine, Kaohsiung Medical University, Kaohsiung 807, Taiwan; ^6^ Department of Biological Sciences and Technology, National University of Tainan, Tainan 700, Taiwan; ^7^ Graduate Institution of Clinical Medical Science, and Graduate Institution of Cancer Biology, China Medical University, Taichung 404, Taiwan; ^8^ Sex Hormone Research Center, China Medical University Hospital, Taichung 404, Taiwan; ^9^ Division of Urology, Department of Surgery, Kaohsiung Veterans General Hospital, Kaohsiung 813, Taiwan; ^10^ Department of Urology, School of Medicine, National Yang-Ming University, Taipei 112, Taiwan; ^11^ Department of Pharmacy, Tajen University, Pingtung 907, Taiwan; ^12^ Department of Urology, National Taiwan University Hospital, College of Medicine, National Taiwan University, Taipei 100, Taiwan; ^13^ Department of Urology, National Taiwan University Hospital Hsin-Chu Branch, Hsinchu 300, Taiwan; ^14^ Department of Pathology, Kaohsiung Medical University Hospital, Kaohsiung 807, Taiwan; ^15^ Department of Pathology, Faculty of Medicine, College of Medicine, Kaohsiung Medical University, Kaohsiung 807, Taiwan; ^16^ Department of Occupational Safety and Health, China Medical University, Taichung 404, Taiwan; ^17^ Department of Pharmacy, China Medical University, Taichung 404, Taiwan; ^18^ Department of Nursing, Asia University, Taichung 413, Taiwan

**Keywords:** progression, prostate cancer, single nucleotide polymorphisms, vitamin D receptor, susceptibility genes

## Abstract

Vitamin D is an important modulator of cellular proliferation through the vitamin D receptor (VDR) that binds to DNA in the regulatory sequences of target genes. We hypothesized that single nucleotide polymorphisms (SNPs) in VDR-binding sites might affect target gene expression and influence the progression of prostate cancer. Using a genome-wide prediction database, 62 SNPs in VDR-binding sites were selected for genotyping in 515 prostate cancer patients and the findings were replicated in an independent cohort of 411 patients. Prognostic significance on prostate cancer progression was assessed by Kaplan-Meier analysis and the Cox regression model. According to multivariate analyses adjusted for known predictors, *HFE* rs9393682 was found to be associated with disease progression for localized prostate cancer, and *TUSC3* rs1378033 was associated with progression for advanced prostate cancer in both cohorts. Vitamin D treatment inhibited *HFE* mRNA expression, and down-regulation of *HFE* by transfecting small interfering RNA suppressed PC-3 human prostate cancer cell proliferation and wound healing ability. In contrast, vitamin D treatment induced *TUSC3* expression, and silencing *TUSC3* promoted prostate cancer cell growth and migration. Further analysis of an independent microarray dataset confirmed that low *TUSC3* expression correlated with poor patient prognosis. Our results warrant further studies using larger cohorts. This study identifies common variants in VDR-binding sites as prognostic markers of prostate cancer progression and *HFE* and *TUSC3* as plausible susceptibility genes.

## INTRODUCTION

Epidemiological studies have demonstrated that low sunlight exposure and poor vitamin D status at higher latitudes account for an elevated risk of a number of cancers, including prostate cancer [1, 2]. Vitamin D can be obtained from the diet; however, it is majorly synthesized in the skin using solar irradiation. The biologically active form of vitamin D_3_, 1α,25-dihydroxyvitamin D_3_ (1,25-VD), is produced by 25-hydroxylase in the liver, followed by 1α-hydroxylase in the kidney. The actions of 1,25-VD are mediated by the vitamin D receptor (VDR), a ligand-activated transcription factor. Upon activation by 1,25-VD, VDR forms a heterodimer with the retinoid X receptor, and binds to vitamin D response elements (VDREs) in the promoters of vitamin D-responsive genes [3]. Prostate cells express VDR and vitamin D metabolizing enzymes, and can respond to 1,25-VD. A volume of data supports multipronged effects of 1,25-VD in the prevention of prostate cancer progression by induction of detoxifying enzymes [4], cell cycle arrest [5], and apoptosis [6], as well as inhibition of prostate cancer cell invasion [7] and angiogenesis [8].

Genetic variants in VDREs may affect VDR-VDRE interactions, thereby resulting in altered expression of target genes and consequent cancer progression. However, no study to date has investigated the single nucleotide polymorphisms (SNPs) in VDR binding sites and their relationship to the clinical outcomes of prostate cancer. Accordingly, we conducted a two-stage study to evaluate the associations of VDRE SNPs with prostate cancer progression, and further assessed the functional relevance of candidate genes of interest, as illustrated in Supplementary Figure 1.

## RESULTS

The clinical characteristics of patients in the discovery and replication cohorts and the association with disease progression are shown in Table 1. For localized prostate cancer, 45 (30.0%) and 75 (43.9%) patients experienced disease progression after radical prostatectomy (RP) during the median follow-up of 23 and 30 months in the discovery and replication cohorts, respectively. Prostate-specific antigen (PSA) at diagnosis, pathologic Gleason score, and pathologic stage were significantly associated with cancer progression in both cohorts. In the advanced prostate cancer group, 271 (74.5%) and 180 (75.3%) patients had disease progression after androgen deprivation therapy (ADT) during the median follow-up of 61 and 57 months in the discovery and replication cohorts, respectively. PSA at ADT initiation, and PSA nadir were significantly associated with cancer progression in both cohorts. Gleason score, clinical stage at diagnosis, and treatment modality were also associated with progression in the discovery cohort, but only weakly associated in the replication cohort.

**Table 1:** Clinical characteristics of study cohorts

Characteristic	Discovery cohort		Replication cohort	
Patients, *N*	515		411	
Age at diagnosis				
Median, y (IQR)	70 (64–77)		72 (66–77)	
PSA at diagnosis				
Median, ng/mL (IQR)	21.6 (9.4–73.0)		21.5 (11.0–81.8)	
Biopsy Gleason score at diagnosis, *N* (%)				
< 7	143 (28.5)		177 (43.8)	
7	213 (42.4)		116 (28.7)	
> 7	146 (29.1)		111 (27.5)	
Clinical stage at diagnosis, *N* (%)				
T1/T2	212 (41.8)		190 (46.3)	
T3/T4/N1	163 (32.1)		129 (31.5)	
M1	132 (26.0)		91 (22.2)	
Localized prostate cancer	Discovery cohort	*P*^a^	Replication cohort	*P*^a^
Patients, *N*	150		171	
Disease progression, *N* (%)				
No	105 (70.0)		96 (56.1)	
Yes	45 (30.0)		75 (43.9)	
Median follow-up time^b^, mo (95% CI)	23 (15–31)		30 (23–37)	
Age at diagnosis				
Median, y (IQR)	65 (61–69)	0.147	67 (62–72)	0.850
PSA at diagnosis				
Median, ng/mL (IQR)	10.4 (6.6–17.0)	0.009	12.7 (8.0–20.8)	< 0.001
Pathologic Gleason score, *N* (%)				
< 7	51 (34.9)	< 0.001	74 (44.0)	< 0.001
7	78 (53.4)		67 (39.9)	
> 7	17 (11.6)		27 (16.1)	
Pathologic stage, *N* (%)				
T1/T2	100 (69.0)	< 0.001	101 (59.1)	< 0.001
T3/T4/N1	45 (31.0)		70 (40.9)	
M1	0 (0.0)		0(0.0)	
Advanced prostate cancer	Discovery cohort	*P*^a^	Replication cohort	*P*^a^
Patients, *N*	365		240	
Disease progression, *N* (%)				
No	93 (25.5)		59 (24.7)	
Yes	271 (74.5)		180 (75.3)	
Median follow-up time^b^, mo (95% CI)	61 (53–69)		57 (45–69)	
Age at diagnosis				
Median, y (IQR)	72 (66–79)	0.520	73 (68–78)	0.034
PSA at ADT initiation				
Median, ng/mL (IQR)	34.2 (10.7–112.0)	0.021	35.6 (11.5–140.7)	0.027
Biopsy Gleason score at diagnosis, *N* (%)				
< 7	92 (25.8)	0.004	103 (43.6)	0.055
7	135 (37.9)		49 (20.8)	
> 7	129 (36.2)		84 (35.6)	
Clinical stage at diagnosis, *N* (%)				
T1/T2	112 (30.9)	0.004	89 (37.2)	0.081
T3/T4/N1	118 (32.6)		59 (24.7)	
M1	132 (36.5)		91 (38.1)	
PSA nadir				
Median, ng/mL (IQR)	0.14 (0.01–1.21)	< 0.001	0.28 (0.01–2.05)	0.002
Treatment modality, *N* (%)				
ADT as primary treatment	153 (42.1)	< 0.001	117 (74.1)	0.073
ADT for post RP PSA failure	44 (12.1)		28 (11.7)	
ADT for post RT PSA failure	7 (1.9)		11 (4.6)	
Neoadjuvant/adjuvant ADT with RT	114 (31.4)		13 (5.4)	
Others	45 (12.4)		10 (4.2)	

Abbreviations: IQR, interquartile range; PSA, prostate-specific antigen; RP, radical prostatectomy; CI, confidence interval; ADT, androgen-deprivation therapy; RT, radiotherapy.

^a^*P* value was calculated by the log-rank test or Cox regression for disease progression.

^b^Median follow-up time and 95% CIs were estimated with the reverse Kaplan-Meier method.

**Figure 1:**
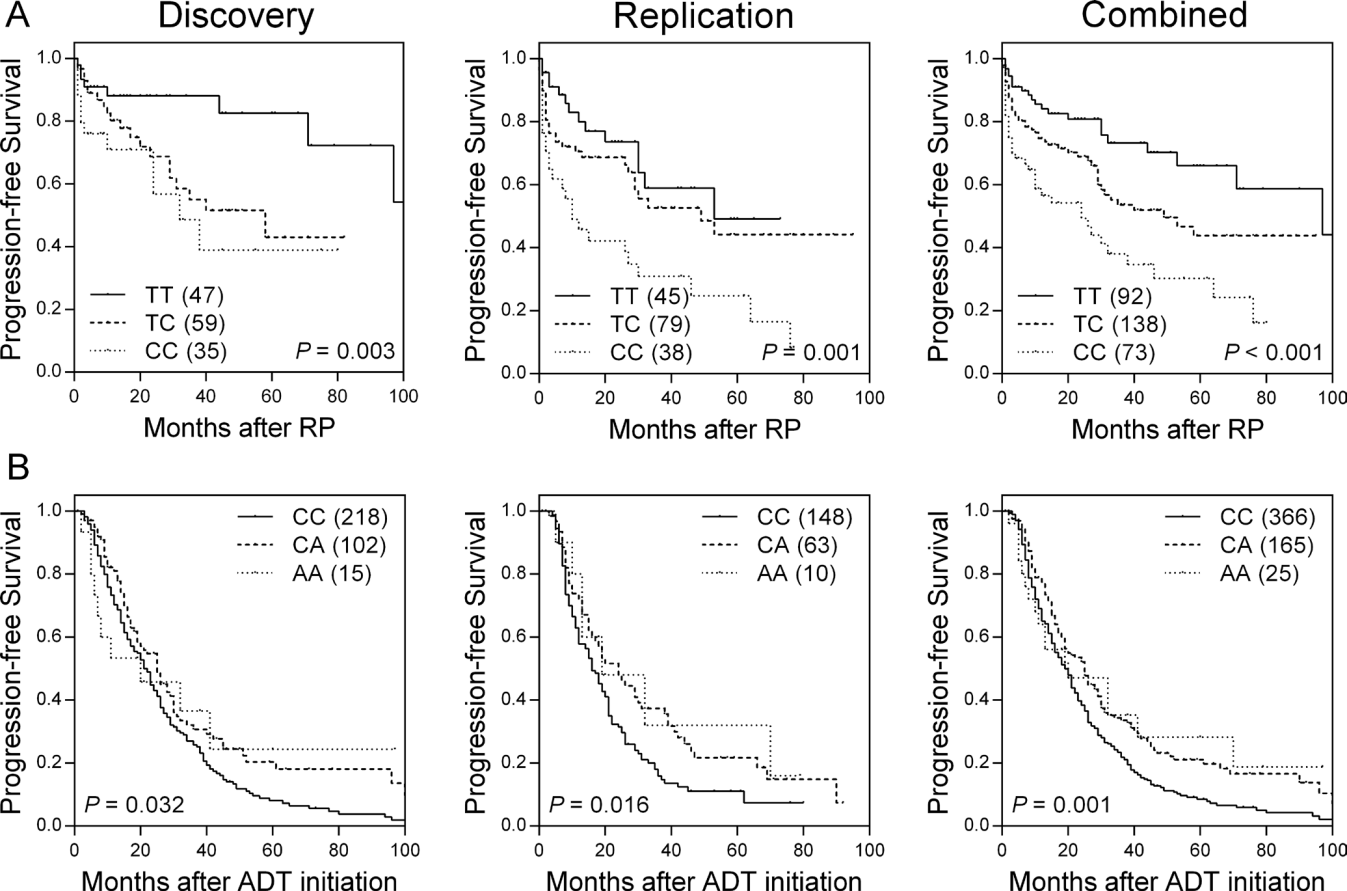
Kaplan-Meier survival curves of progression-free survival by **(A)** rs9393682 genotypes for localized prostate cancer patients undergoing RP, and **(B)** rs1378033 genotypes for advanced prostate cancer patients undergoing ADT, in discovery cohort (left), replication cohort (middle), and combined analysis (right).

Of the 62 SNPs in VDREs analyzed in the discovery cohort, six SNPs were associated with time to progression (TTP) (Supplementary Table 1) in localized prostate cancer patients. rs9393682 was found to be significantly associated with TTP in the same direction as the discovery cohort in an independent replication cohort. In combined analysis, rs9393682 was associated with a per-allele hazard ratio (HR) of 1.79 [95% confidence interval (CI), 1.38–2.33; *P* < 0.001; Table 2 and Figure 1A]. This association remained significant (*P* = 0.006) after adjusting for age, PSA at diagnosis, pathologic Gleason score, and stage. Furthermore, the outcome prediction model based on clinical factors (age, PSA at diagnosis, pathologic Gleason score, and stage) plus rs9393682 was significantly improved over the model with clinical factors only, as indicated by the likelihood ratio test (χ^2^ 69.63, df 1, *P* < 0.001).

**Table 2:** Association of rs9393682 with disease progression in localized prostate cancer patients treated with RP

SNP	Univariate analysis										Multivariate analysisa					
Genotype	Discovery				Replication				Combined		Discovery		Replication		Combined	
	*N*	Prog	HR (95% CI)	*P*	*N*	Prog	HR (95% CI)	*P*	HR (95% CI)	*P*	HR (95% CI)	*P*	HR (95% CI)	*P*	HR (95% CI)	*P*
rs9393682																
TT	47	8	1.00		45	14	1.00		1.00		1.00		1.00		1.00	
TC	59	20	**2.65 (1.11-6.29)**	**0.03**	79	31	1.37 (0.73-2.59)	0.33	**1.72 (1.03-2.86)**	**0.04**	1.65 (0.57-4.79)	0.36	1.23 (0.63-2.41)	0.55	1.34 (0.76-2.36)	0.31
CC	35	13	**3.82 (1.51-9.63)**	**0.005**	38	26	**2.83 (1.47-5.43)**	**0.002**	**3.13 (1.83-5.34)**	**< 0.001**	**3.53 (1.21-10.3)**	**0.02**	1.76 (0.88-3.51)	0.11	**2.16 (1.21-3.87)**	**0.009**
TC/CC vs TT			**3.01 (1.32-6.84)**	**0.009**			1.79 (1.00-3.23)	0.05	**2.13 (1.32-3.42)**	**0.002**	2.26 (0.84-6.10)	0.11	1.43 (0.77-2.66)	0.26	1.63 (0.96-2.75)	0.07
CC vs TT/TC			**2.05 (1.05-3.98)**	**0.04**			**2.30 (1.42-3.73)**	**0.001**	**2.21 (1.50-3.27)**	**< 0.001**	**2.50 (1.21-5.15)**	**0.01**	1.53 (0.91-2.58)	0.11	**1.81 (1.18-2.76)**	**0.006**
Trend			**1.87 (1.23-2.86)**	**0.004**			**1.74 (1.24-2.42)**	**0.001**	**1.79 (1.38-2.33)**	**< 0.001**	**1.94 (1.15-3.26)**	**0.01**	1.34 (0.95-1.89)	0.10	**1.50 (1.12-2.00)**	**0.006**

Abbreviations: RP, radical prostatectomy; SNP, single nucleotide polymorphism; Prog, progression; HR, hazard ratio; 95% CI, 95% confidence interval; PSA, prostate-specific antigen.

^a^Adjusted by age, PSA at diagnosis, pathologic Gleason score, and pathologic stage.

*P* < 0.05 are in boldface.

For the advanced prostate cancer group, four VDRE SNPs were associated with TTP during ADT (Supplementary Table 2). Only rs1378033 showed significant correlation with a decreased risk of disease progression in both the discovery and replication cohorts, and upon combined analysis (HR 0.75, 95% CI 0.63–0.89, *P* = 0.001; Table 3 and Figure 1B). After adjusting for known predictors, the association remained significant (*P* = 0.004). The model based on clinical factors plus rs1378033 was significantly improved over the model with clinical factors only, as indicated by the likelihood ratio test (χ^2^ 380.51, df 1, *P* < 0.001).

**Table 3:** Association of rs1378033 with disease progression in advanced prostate cancer patients treated with ADT

SNP	Univariate analysis										Multivariate analysisa					
Genotype	Discovery				Replication				Combined		Discovery		Replication		Combined	
	*N*	Prog	HR (95% CI)	*P*	*N*	Prog	HR (95% CI)	*P*	HR (95% CI)	*P*	HR (95% CI)	*P*	HR (95% CI)	*P*	HR (95% CI)	*P*
rs1378033																
CC	218	172	1.00		148	113	1.00		1.00		1.00		1.00		1.00	
CA	102	69	**0.72 (0.55-0.96)**	**0.02**	63	48	**0.66 (0.47-0.94)**	**0.02**	**0.70 (0.56-0.86)**	**<0.001**	**0.70 (0.52-0.95)**	**0.02**	**0.68 (0.47-0.98)**	**0.04**	**0.69 (0.55-0.87)**	**0.002**
AA	15	10	0.78 (0.41-1.47)	0.44	10	7	0.63 (0.29-1.36)	0.24	0.72 (0.44-1.17)	0.18	0.74 (0.39-1.44)	0.38	0.69 (0.32-1.51)	0.35	0.72 (0.44-1.18)	0.19
CA/AA vs CC			**0.73 (0.56-0.95)**	**0.02**			**0.66 (0.47-0.92)**	**0.01**	**0.70 (0.57-0.87)**	**<0.001**	**0.71 (0.53-0.94)**	**0.02**	**0.68 (0.48-0.96)**	**0.03**	**0.70 (0.56-0.87)**	**0.002**
AA vs CC/CA			0.87 (0.46-1.63)	0.66			0.73 (0.34-1.56)	0.42	0.81 (0.50-1.32)	0.40	0.82 (0.42-1.57)	0.54	0.79 (0.37-1.71)	0.55	0.81 (0.49-1.33)	0.40
Trend			**0.78 (0.62-0.98)**	**0.04**			**0.71 (0.54-0.95)**	**0.02**	**0.75 (0.63-0.89)**	**0.001**	**0.77 (0.60-0.98)**	**0.03**	**0.74 (0.55-0.99)**	**0.04**	**0.76 (0.63-0.92)**	**0.004**

Abbreviations: ADT, androgen-deprivation therapy; SNP, single nucleotide polymorphism; Prog, progression; HR, hazard ratio; 95% CI, 95% confidence interval; PSA, prostate-specific antigen.

^a^Adjusted by age, PSA at ADT initiation, biopsy Gleason score, clinical stage, PSA nadir, and treatment modality.

*P* < 0.05 are in boldface.

**Figure 2:**
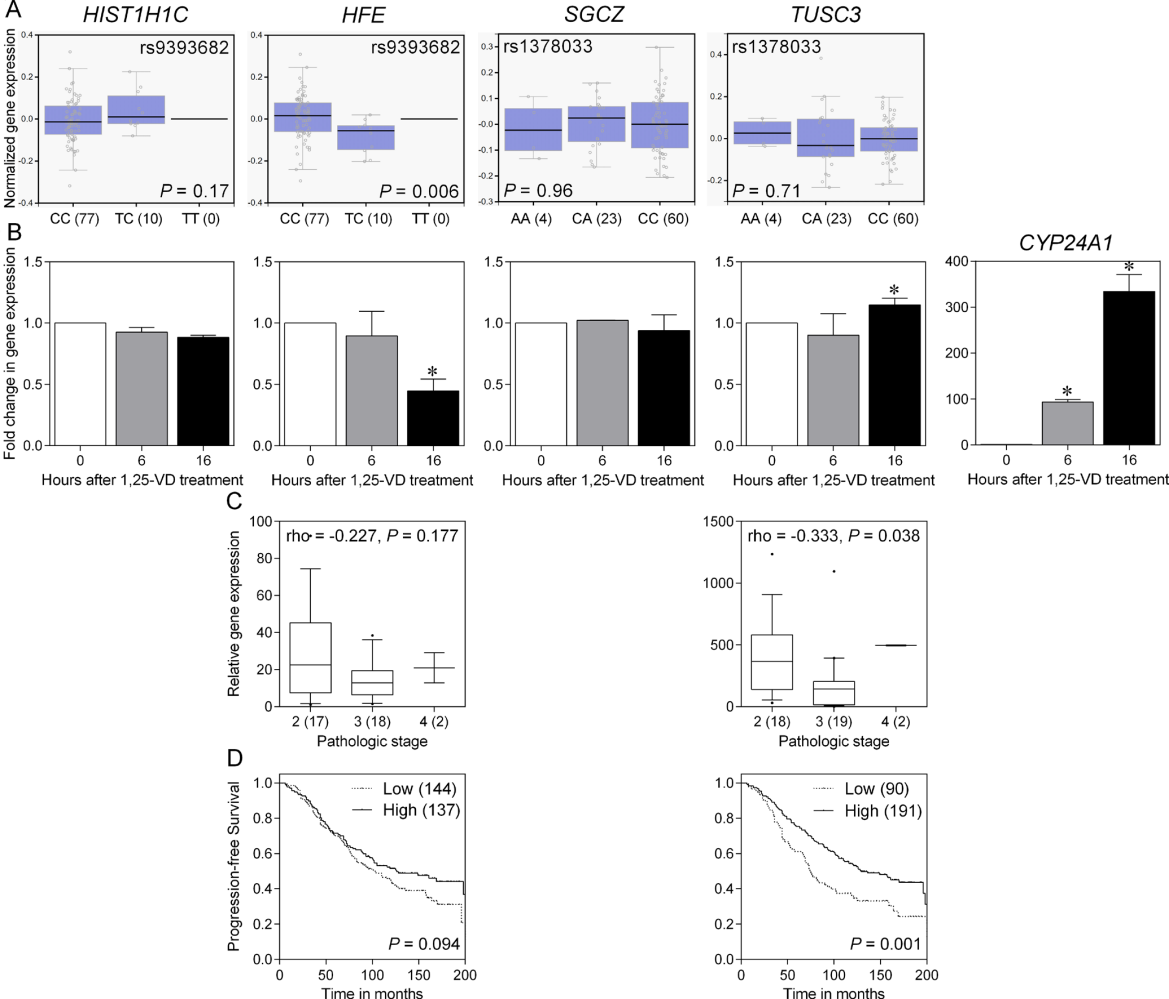
Functional analysis of SNPs and candidate genes associated with prostate cancer progression (**A**) Expression quantitative trait locus associations of rs9393682 and rs1378033 with nearby genes (*HIST1H1C* and *HFE* for rs9393682; *SGCZ* and *TUSC3* for rs1378033) expression in prostate tissues (GTEx dataset). Numbers in parentheses indicate the number of cases. (**B**) Effect of 1,25-VD on candidate genes expression. PC-3 cells were treated with vehicle or 1,25-VD for indicated time points. Total mRNA was prepared and the corresponding gene expression was determined by qRT-PCR. Values represent the fold change in gene expression relative to vehicle-treated control. Asterisk indicates significant difference (*P* < 0.05). (**C**) Correlation of candidate genes expression with prostate cancer aggressiveness. Numbers in parentheses indicate the number of cases. (**D**) Kaplan-Meier analysis of progression-free survival based on candidate genes expression using an independent dataset from Sboner *et al.* Numbers in parentheses indicate the number of cases.

rs9393682 is located in the intergenic region between *HIST1H1C* (histone cluster 1 H1 family member c) and *HFE* (hemochromatosis), and rs1378033 is located in the intron of *SGCZ* (sarcoglycan zeta) and 5′ of *TUSC3* (tumor suppressor candidate 3). To gain initial insight for further analysis, we investigated if rs9393682 and rs1378033 were associated with differential expression of nearby genes expression in prostate tissues. The Genotype-Tissue Expression (GTEx) database showed a significant trend for increased *HFE* expression in rs9393682 C allele carriers (*P* = 0.006; Figure 2A). rs9393682 and rs1378033 fall within putative VDREs, and may alter 1,25-VD-mediated gene regulation. The effects of 1,25-VD on the mRNA expression levels of *HIST1H1C*, *HFE*, *SGCZ*, *TUSC3*, and *CYP24A1* (cytochrome P450 family 24 subfamily A member 1), a well-known 1,25-VD target gene, were examined using quantitative real-time polymerase chain reaction (qRT-PCR) in PC-3 human prostate cancer cells. As a positive control, 1,25-VD can strongly induce *CYP24A1* expression (Figure 2B). The expression of *TUSC3* was also induced by 1,25-VD, but *HFE* was downregulated. We then assessed *HFE* and *TUSC3* expression using a prostate cancer complementary DNA array containing 39 tissue samples. A significant downregulation of *TUSC3* expression was observed in more advanced stage cancer samples (*P* = 0.038, Figure 2C). The prognostic values of *HFE* and *TUSC3* on prostate cancer progression were further evaluated using a publicly available prostate cancer microarray dataset. Patients were dichotomized by *HFE* or *TUSC3* gene expression using an optimization algorithm for the minimum *P* value. Low expression of *TUSC3* was associated with shorter progression-free survival (*P* = 0.001; Figure 2D). Taken together, these observations indicate that 1,25-VD can stimulate *TUSC3* expression, and down-regulation of *TUSC3* in late stages of cancer may increase the risk of prostate cancer progression.

**Figure 3:**
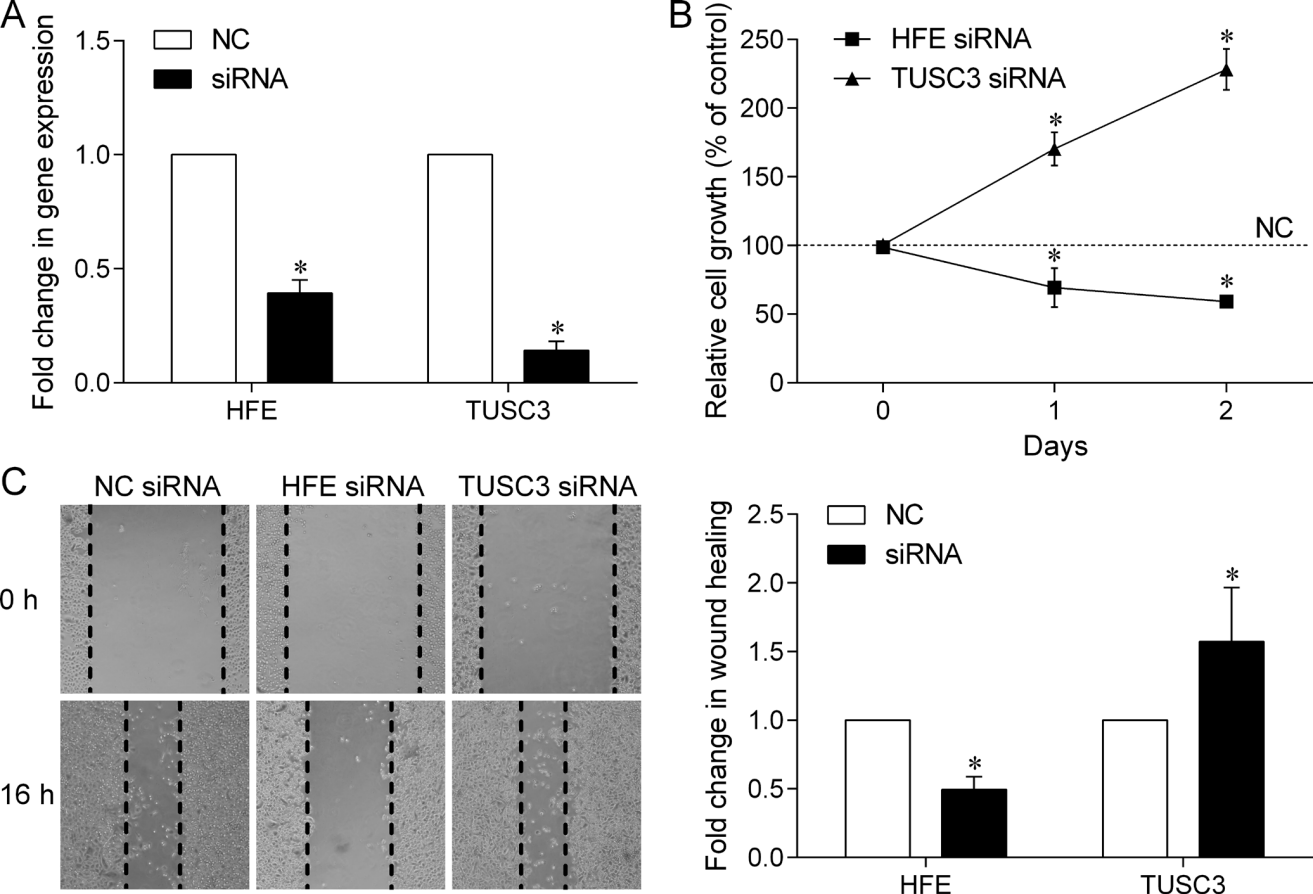
Effect of down-regulated *HFE* and *TUSC3* expression on prostate cancer cell growth and migration (**A**) The mRNA expression of *HFE* and *TUSC3* was effectively down-regulated by transfecting corresponding siRNAs into PC-3 human prostate cancer cells. (**B**) Cell growth assay showed that silencing *HFE* expression could inhibit PC-3 cell proliferation; however, silencing *TUSC3* expression could promote cell proliferation. (**C**) Down-regulation of *HFE* decreases the ability of prostate cancer cells to migrate; however, down-regulation of *TUSC3* increases their ability to migrate. The scratch wound healing assay was carried out for 16 h after siRNA transfection. The values are the average of at least three independent experiments where asterisk indicates *P* < 0.05. NC, negative control.

To better understand the roles of *HFE* and *TUSC3* in prostate cancer, we used small interfering RNAs (siRNAs) to silence their expression in PC-3 cells, and evaluate the consequences of loss of *HFE* or *TUSC3* function on cell proliferation and migration. As shown in Figure 3A, *HFE* or *TUSC3* gene expression was effectively down-regulated by corresponding siRNAs, compared to that of the negative control (NC)-siRNA. Silencing of *HFE* inhibited cell proliferation, whereas knockdown of *TUSC3* in PC-3 cells demonstrated a significant growth advantage over NC cells (Figure 3B). In the wound healing assay, our data showed that *HFE* silencing retarded wound closure by 50%, but *TUSC3* silenced PC-3 cells increased migration ability by 157% (Figure 3C). These data demonstrated that both *HFE* and *TUSC3* could regulate prostate cancer cell proliferation and migration.

## DISCUSSION

We conducted a two-stage study to identify genetic variants in VDR binding sites corresponding with prognosis of patients with surgically resected localized prostate cancer and patients with ADT treated advanced disease. The associations of *HFE* rs9393682 and *TUSC3* rs1378033 with disease progression were replicated across both stages of the study for localized and advanced prostate cancer, respectively. In addition, knockdown *HFE* and *TUSC3* expression significantly influenced prostate cancer cell proliferation and migration. We also found that *TUSC3* expression could be induced by 1,25-VD, and its expression was down-regulated in advanced stage cancer. These findings suggest that *TUSC3* could be used as a prognostic marker for prostate cancer.

Based on the HaploReg data, rs9393682 and its correlated variants within a linkage disequilibrium block are positioned in the promoter elements, and rs1378033 is situated in a regulatory region containing DNase hypersensitivity peaks in different cell lines. The expression of two genes near these VDRE SNPs, *HFE* and *TUSC3*, can be regulated by 1,25-VD, suggesting that *HFE* and *TUSC3* might be potential vitamin D target genes and thus contribute to prostate cancer progression. HFE has been recognized as a receptor for β2-microglobulin (β2-M), and β2-M is a known growth-promoting gene for several human cancers, including prostate cancer [9, 10]. β2-M interacts with HFE to modulate intracellular iron, activate iron responsive hypoxia inducible factor-1α signaling, and promote cancer bone metastasis through its induction of epithelial-mesenchymal transition in cancer cells [11]. Consistent with these studies, our data showed that silencing *HFE* expression could markedly inhibit prostate cancer cell growth and migration (Figure 3). Although no association was observed between *HFE* expression and prostate cancer stage and progression (Figure 2C and 2D), high *HFE* expression was significantly associated with shorter overall survival in multiple The Cancer Genome Atlas (TCGA) cancer sets, such as breast invasive carcinoma, brain lower grade glioma, and pancreatic adenocarcinoma (Supplementary Figure 2; prostate adenocarcinoma was not calculated because of low number of deaths). This discrepancy may be due to the relative small sample size of tumor tissues used to assess the association between gene expression and patient prognosis. TUSC3 was identified as a potential tumor suppressor gene on chromosome 8p22, a common homozygously deleted region of the metastatic prostate cancer [12]. It has been described as a homologue of a subunit of the yeast oligosaccharyltransferase complex, which modulates glycosylation of proteins in the endoplasmic reticulum (ER) [13]. Loss of TUSC3 has been proposed to facilitate prostate cancer progression by increasing protein glycosylation, alleviating unfolded protein response and ER stress, and promoting Akt survival signaling [14]. Our data also showed that silencing *TUSC3* expression could increase prostate cancer cell proliferation and migration (Figure 3). In addition, *TUSC3* expression was down-regulated in late stage cancers, and low *TUSC3* expression was significantly associated with shorter TTP in prostate cancer, as well as decreased survival in several TCGA cancer sets (Supplementary Figure 2). However, no SNP is simultaneously associated with disease progression in both localized and advanced prostate cancers, suggesting that distinct biological pathways might be involved in different stages of the disease and the treatments adopted.

The associations of *HFE* rs9393682 and *TUSC3* rs1378033 with prostate cancer progression was replicated across both cohorts, which would reduce false-positive findings in this study. In addition, our functional studies support these gene-disease associations. However, several limitations in the present study should be considered. First, we chose the TTP end point based on serum PSA, due to its biologic and clinical relevance. A rising PSA is often the first indication of the development of progressive disease and precipitates a change in therapy. Thus, the end point of PSA TTP most closely identifies the timing of treatment failure. Second, the modest sample size of both cohorts did not have optimal statistical power for discovering and replicating the association, so the observed *P* values did not reach a level that would avoid false-positives arising from multiple testing. Third, although we speculated that VDRE SNPs might affect VDR binding to genomic sequences, the specific mechanism was not clear. However, the two susceptibility genes identified in this study, *HFE* and *TUSC3*, can indeed be regulated by vitamin D. Finally, our findings in this homogeneous Chinese Han population might not be applicable to other ethnic groups. Further functional characterizations and studies with larger patient cohorts are required to validate our findings.

This study shows that *HFE* rs9393682 and *TUSC3* rs1378033 influence TTP in patients with prostate cancer. Silencing *TUSC3* promotes prostate cancer cell proliferation and migration, and its expression is decreased in advanced stage cancer tissues, as well as in patients with poor prognosis. These results suggest that *TUSC3* may function as a candidate susceptibility gene, and is a promising target for prostate cancer progression.

## MATERIALS AND METHODS

### Patient recruitment and data collection

The study included 926 patients with prostate cancer divided into two independent cohorts (Table 1). The discovery cohort comprised 150 men with localized prostate cancer who underwent RP as initial therapy, and 365 men with advanced cancer on ADT from the National Taiwan University Hospital located in northern Taiwan, as described previously [15–17]. The replication cohort was composed of 171 patients with localized prostate cancer, and 240 patients with advanced disease from the Kaohsiung Medical University Hospital, E-Da Hospital, and Kaohsiung Veterans General Hospital, all located in southern Taiwan. Detailed clinical information was obtained from patients’ medical records. The primary outcome variable was TTP. TTP after RP for localized prostate cancer was defined as two consecutive PSA measurements of more than 0.2 ng/mL at an interval of more than three months, and the first of the consecutive rises was considered as the time of progression [18]. TTP after ADT for advanced prostate cancer was defined as a serial rise in PSA, at least 2 rises in PSA (> 1 week apart), greater than the PSA nadir [19]. Initiation of secondary hormone treatment for rising PSA was also considered as a progression event. This study was approved by the Institutional Review Board of Kaohsiung Medical University Hospital. Written informed consent was obtained from each patient, and the study was carried out in accordance with approved guidelines.

### SNP selection and genotyping

Since transcription factors are known to regulate different genes in different cellular contexts [20], we used a genome-wide cis-regulatory module prediction database, PReMod (genomequebec.mcgill.ca/PReMod) [21], to identify putative VDREs in the whole human genome instead of the chromatin immunoprecipitation data. The PReMod algorithm predicts that a total of 11,342 sites within the human genome are bound by the VDR (canonical VDR position weight matrix: M00444; consensus: GGGKNARNRRGGWSA) [22]. We identified SNPs within VDREs by comparing two hexameric half-sites of these putative VDREs with HapMap SNPs CHB (Han Chinese, Beijing, China) data in the UCSC table browser (NCBI35/hg17) [23, 24]. SNPs with a minor allele frequency less of than 0.10 in the HapMap CHB population were excluded, thus leaving 68 SNPs in VDREs that were initially selected for analysis. The threshold of 0.10 was chosen because it was considered to be the lowest minor allele frequency for a SNP with a relative risk of at least two being detectable with a sample size of 500.

Genomic DNA was extracted from patients’ peripheral blood using the QIAamp DNA Blood Mini Kit (Qiagen, Valencia, CA) and stored at −80 °C until the time of the study. Genotyping was performed as described previously [18] at the National Center for Genome Medicine, Academia Sinica, Taiwan, using Agena Bioscience iPLEX matrix-assisted laser desorption/ionization time-of-flight mass-spectrometry technology. The average genotype call rate for these SNPs was 95.2%. Ten samples were blindly duplicated for quality control and the genotype concordance was 99.7%. Six SNPs that significantly deviated from the Hardy-Weinberg equilibrium (*P* < 0.005) were removed, leaving 62 SNPs for further statistical analysis.

### qRT-PCR analysis, lentiviral transduction, cell proliferation and wound healing assays, and bioinformatics analysis

PC-3 (CRL-1435) human prostate cancer cell line was purchased from ATCC (Manassas, VA) and maintained in the recommended culture media. The identity of the cell line was checked by Cell ID System and Promega GenePrint 10 System through short tandem repeat analysis (Mission Biotech, Taipei, Taiwan). A comprehensive methods section is available in Supplementary Methods.

### Statistical analysis

Patient clinicopathologic characteristics were summarized as number and percentage of patients or median and interquartile range of values. The association between patient characteristics with TTP was assessed by the log-rank test or Cox regression analysis. Individual SNPs were first assessed by association with TTP using the log-rank test under dominant, recessive, and additive models because the function of the SNPs was unknown. Cox proportional hazards regression was then conducted on each SNP as an isolated covariate with adjustment for known prognostic factors. In localized prostate cancer, multiple explanatory variables included age, PSA at diagnosis, pathologic Gleason score, and stage, as previously defined [25]. In advanced prostate cancer, multiple explanatory variables included age, PSA at ADT initiation, biopsy Gleason score, clinical stage, PSA nadir, and treatment modality, as previously defined [26]. Heterogeneity between cohorts was evaluated by Cochran’s *χ*^2^-based *Q* statistical test. If the results of the *Q* test were significant, a random-effects model was used to accommodate the diversity; otherwise, the combined HR was estimated using the fixed-effects model. The Statistical Package for the Social Sciences software version 22.0.0 (IBM, Armonk, NY) was used for statistical analyses. A two-sided *P* value of < 0.05 was considered statistically significant.

## SUPPLEMENTARY MATERIALS FIGURES AND TABLES







## References

[1] Garland CF, Garland FC (1980). Do sunlight and vitamin D reduce the likelihood of colon cancer?. Int J Epidemiol.

[2] Schwartz GG, Hulka BS (1990). Is vitamin D deficiency a risk factor for prostate cancer? (Hypothesis). Anticancer Res.

[3] Kliewer SA, Umesono K, Noonan DJ, Heyman RA, Evans RM (1992). Convergence of 9-cis retinoic acid and peroxisome proliferator signalling pathways through heterodimer formation of their receptors. Nature.

[4] Bao BY, Ting HJ, Hsu JW, Lee YF (2008). Protective role of 1 alpha, 25-dihydroxyvitamin D3 against oxidative stress in nonmalignant human prostate epithelial cells. Int J Cancer.

[5] Bao BY, Hu YC, Ting HJ, Lee YF (2004). Androgen signaling is required for the vitamin D-mediated growth inhibition in human prostate cancer cells. Oncogene.

[6] Hsieh T, Wu JM (1997). Induction of apoptosis and altered nuclear/cytoplasmic distribution of the androgen receptor and prostate-specific antigen by 1alpha,25-dihydroxyvitamin D3 in androgen-responsive LNCaP cells. Biochem Biophys Res Commun.

[7] Bao BY, Yeh SD, Lee YF (2006). 1alpha,25-dihydroxyvitamin D3 inhibits prostate cancer cell invasion via modulation of selective proteases. Carcinogenesis.

[8] Bao BY, Yao J, Lee YF (2006). 1alpha, 25-dihydroxyvitamin D3 suppresses interleukin-8-mediated prostate cancer cell angiogenesis. Carcinogenesis.

[9] Gross M, Top I, Laux I, Katz J, Curran J, Tindell C, Agus D (2007). Beta-2-microglobulin is an androgen-regulated secreted protein elevated in serum of patients with advanced prostate cancer. Clin Cancer Res.

[10] Teasdale C, Mander AM, Fifield R, Keyser JW, Newcombe RG, Hughes LE (1977). Serum beta2-microglobulin in controls and cancer patients. Clin Chim Acta.

[11] Josson S, Nomura T, Lin JT, Huang WC, Wu D, Zhau HE, Zayzafoon M, Weizmann MN, Gururajan M, Chung LW (2011). beta2-microglobulin induces epithelial to mesenchymal transition and confers cancer lethality and bone metastasis in human cancer cells. Cancer Res.

[12] Bova GS, MacGrogan D, Levy A, Pin SS, Bookstein R, Isaacs WB (1996). Physical mapping of chromosome 8p22 markers and their homozygous deletion in a metastatic prostate cancer. Genomics.

[13] Kelleher DJ, Karaoglu D, Mandon EC, Gilmore R (2003). Oligosaccharyltransferase isoforms that contain different catalytic STT3 subunits have distinct enzymatic properties. Mol Cell.

[14] Horak P, Tomasich E, Vanhara P, Kratochvilova K, Anees M, Marhold M, Lemberger CE, Gerschpacher M, Horvat R, Sibilia M, Pils D, Krainer M (2014). TUSC3 loss alters the ER stress response and accelerates prostate cancer growth *in vivo*. Sci Rep.

[15] Bao BY, Pao JB, Huang CN, Pu YS, Chang TY, Lan YH, Lu TL, Lee HZ, Juang SH, Chen LM, Hsieh CJ, Huang SP (2011). Polymorphisms inside microRNAs and microRNA target sites predict clinical outcomes in prostate cancer patients receiving androgen-deprivation therapy. Clin Cancer Res.

[16] Huang CY, Huang SP, Lin VC, Yu CC, Chang TY, Lu TL, Chiang HC, Bao BY (2015). Genetic variants of the autophagy pathway as prognostic indicators for prostate cancer. Sci Rep.

[17] Huang SP, Huang LC, Ting WC, Chen LM, Chang TY, Lu TL, Lan YH, Liu CC, Yang WH, Lee HZ, Hsieh CJ, Bao BY (2009). Prognostic significance of prostate cancer susceptibility variants on prostate-specific antigen recurrence after radical prostatectomy. Cancer Epidemiol Biomarkers Prev.

[18] Huang SP, Ting WC, Chen LM, Huang LC, Liu CC, Chen CW, Hsieh CJ, Yang WH, Chang TY, Lee HZ, Bao BY (2010). Association analysis of Wnt pathway genes on prostate-specific antigen recurrence after radical prostatectomy. Ann Surg Oncol.

[19] Ross RW, Oh WK, Xie W, Pomerantz M, Nakabayashi M, Sartor O, Taplin ME, Regan MM, Kantoff PW, Freedman M (2008). Inherited variation in the androgen pathway is associated with the efficacy of androgen-deprivation therapy in men with prostate cancer. J Clin Oncol.

[20] Zeitlinger J, Simon I, Harbison CT, Hannett NM, Volkert TL, Fink GR, Young RA (2003). Program-specific distribution of a transcription factor dependent on partner transcription factor and MAPK signaling. Cell.

[21] Ferretti V, Poitras C, Bergeron D, Coulombe B, Robert F, Blanchette M (2007). PReMod: a database of genome-wide mammalian cis-regulatory module predictions. Nucleic Acids Res.

[22] Roche PJ, Hoare SA, Parker MG (1992). A consensus DNA-binding site for the androgen receptor. Mol Endocrinol.

[23] Karolchik D, Hinrichs AS, Furey TS, Roskin KM, Sugnet CW, Haussler D, Kent WJ (2004). The UCSC Table Browser data retrieval tool. Nucleic Acids Res.

[24] Thorisson GA, Smith AV, Krishnan L, Stein LD (2005). The International HapMap Project Web site. Genome Res.

[25] Huang SP, Levesque E, Guillemette C, Yu CC, Huang CY, Lin VC, Chung IC, Chen LC, Laverdiere I, Lacombe L, Fradet Y, Chang TY, Lee HZ (2014). Genetic variants in microRNAs and microRNA target sites predict biochemical recurrence after radical prostatectomy in localized prostate cancer. Int J Cancer.

[26] Huang CN, Huang SP, Pao JB, Chang TY, Lan YH, Lu TL, Lee HZ, Juang SH, Wu PP, Pu YS, Hsieh CJ, Bao BY (2012). Genetic polymorphisms in androgen receptor-binding sites predict survival in prostate cancer patients receiving androgen-deprivation therapy. Ann Oncol.

