# Supplementary material for: Vitamin D receptor-binding site variants affect prostate cancer progression
Source: Oncotarget. 2017 May 26;8(43):74119–28. doi: 10.18632/oncotarget.18271 (PMC5650327; doi:10.18632/oncotarget.18271)
Supplement: Supplementary file 1 [file oncotarget-08-74119-s001.pdf]

Vitamin D receptor-binding site variants affect prostate cancer progression

SUPPLEMENTARY MATERIALS

Quantitative real-time polymerase chain reaction (qRT-PCR) analysis

PC-3 human prostate cancer cells were treated with ethanol vehicle or 100 nM 1,25-VD for the indicated time points, then total RNA was harvested by Trizol (Invitrogen, Carlsbad, CA). We carried out reverse transcription with the Super Script II kit (Invitrogen) and PCR amplifications with SYBR Green PCR Master Mix on an Applied Biosystems StepOnePlus Real-Time PCR System (Applied Biosystems, Foster City, CA) according to the manufacturer's instructions. The quantification of each sample was normalized using 18S rRNA. Primers used were *HIST1H1C*: 5′-ACACCgAAgAAAgCgAAgAA-3′ (forward) and 5′-AgCCTTAgCAgCACTTTTgg-3′ (reverse); *HFE*: 5′-CgTCTggCACCCTAgTCATTg-3′ (forward) and 5′-CATggCTCCTCTTgAACCCTg-3′ (reverse); *SGCZ*: 5′-TCTTTgTCCTTCTgCTgTTgg-3′ (forward) and 5′-AgTCggATTCCCTTCTTggT-3′ (reverse); *TUSC3*: 5′-gAACTCCTggCgCTATTCATC-3′ (forward) and 5′-TCTCTTAggTCTgCCTTTTggA-3′ (reverse); *CYP24A1*: 5′-CAGCAGCATCTCATCTACC-3′ (forward) and 5′-GACAGCCTCAGAGCATTG-3′ (reverse); *18S*: 5′-GCGCAAATTACCCACTCCCG-3′ (forward) and 5′-CCCGCTCCCAAGATCCAACT-3′ (reverse).

The expression levels of *HFE*, *TUSC3*, and *β-actin* (*ACTB*) were measured using the TissueScan human prostate cancer cDNA array III, including 39 prostate cancer samples (OriGene Technologies, Rockville, MD). qRT-PCR was performed using prevalidated TaqMan gene expression assays (Applied Biosystems), *HFE* (Hs00373474_m1), *TUSC3* (Hs04234982_m1), and *ACTB* (Hs01060665_g1), on the Applied Biosystems StepOnePlus Real-Time PCR System. The quantification of each sample was normalized using the housekeeping gene *ACTB*.

Lentiviral transduction

Down-regulation of *HFE* and *TUSC3* in PC-3 human prostate cancer cells was performed by lentiviral delivery using a pLKO.1 vector containing scramble small interfering RNA (siRNA), *HFE* siRNA (Clone ID, TSCN0000060022), and *TUSC3* siRNA (Clone ID, TSCN0000299107) (National RNAi Core Facility Platform located at the institute of Molecular Biology/ Genome Research Center, Academia Scinica, supported by the National Core Facility Program for Biotechnology).

Cell proliferation and wound healing assays

Cell viability was assessed at 0, 1, and 2 days using WST-1 reagent (Roche Applied Science, Mannheim, Germany) according to the manufacturer's instructions. Briefly, 24 h after transfection, PC-3 cells were digested and seeded into 96-well plates at a density of 1000 cells/ well. Then, 10 μL of WST-1 solution was added into each well, and cells were allowed to incubate for 1 h at 37 °C. Cell viability was then quantified by colorimetric detection using the PARADIGM microplate detection platform (Beckman Coulter, Indianapolis, IN) at an absorbance of 450 nm and 690 nm, to generate an optical density proportional to the relative abundance of live cells in the given wells. For the wound healing cell migration assay, 2×10^5 transfected PC-3 cells were seeded into 12-well plates and allowed to adhere for 18 h. A 200-μL pipette tip was then used to create a linear wound area. Wound closure was observed and photographed over the course of 16 h. Cell migration ability was defined by subtracting the wound area (mm^2) at 0 h from that at 16 h. The photographic images were analyzed using NIS Elements BR3.1v software (Nikon, Tokyo, Japan).

Bioinformatics analysis

We used several bioinformatics tools to assess whether VDRE SNPs or linked genetic variants were associated with a putative function that might affect patient outcomes. Genotype-Tissue Expression (GTEx) data were used to correlate the relationships between SNPs and gene expression levels in human prostate tissues [1]. HaploReg v4.1 [2] was accessed to identify

the regulatory potential of the region near these SNPs. To further validate our findings, the association of *HFE* and *TUSC3* expression with prostate cancer progression was analyzed using publicly available microarray data from Sboner *et al*. [3], and the associations with overall survival in breast invasive carcinoma, brain lower grade glioma, and pancreatic adenocarcinoma were analyzed using The Cancer Genome Atlas (TCGA) datasets (http://cancergenome.nih.gov/).

SUPPLEMENTARY REFERENCES

1. The Genotype-Tissue Expression (GTEx) project. Nat Genet. 2013; 45:580–585.
2. Ward LD and Kellis M. HaploReg: a resource for exploring chromatin states, conservation, and regulatory motif alterations within sets of genetically linked variants. Nucleic Acids Res. 2012; 40:D930–934.
3. Sboner A, Demichelis F, Calza S, Pawitan Y, Setlur SR, Hoshida Y, Perner S, Adami HO, Fall K, Mucci LA, Kantoff PW, Stampfer M, Andersson SO, et al. Molecular sampling of prostate cancer: a dilemma for predicting disease progression. BMC Med Genomics. 2010; 3:8.

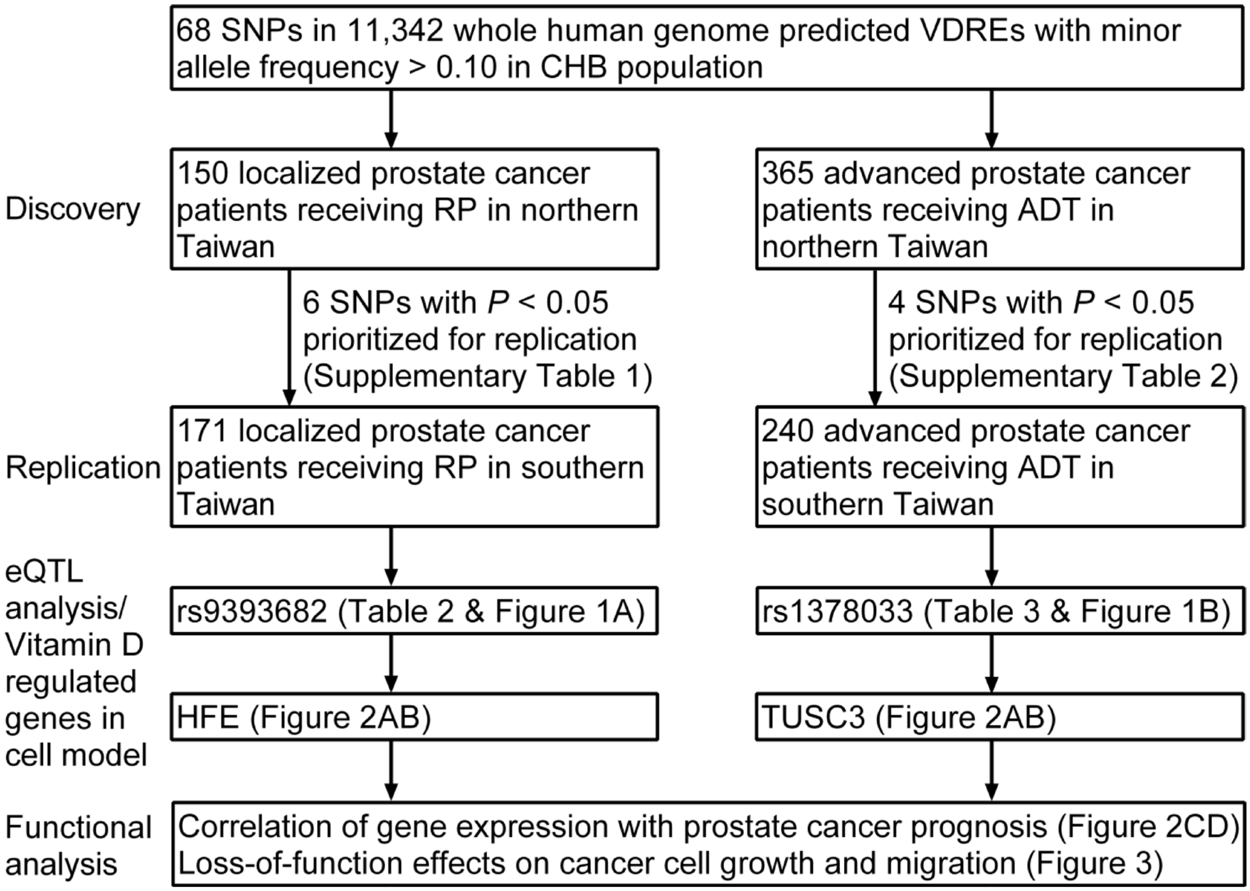

Supplementary Figure 1: Study design and key findings. The discovery stage 1, replication stage 2, and subsequent functional analysis are illustrated. Two candidate susceptibility genes, *HFE* and *TUSC3*, for prostate cancer progression were identified after replication testing and functional analysis.

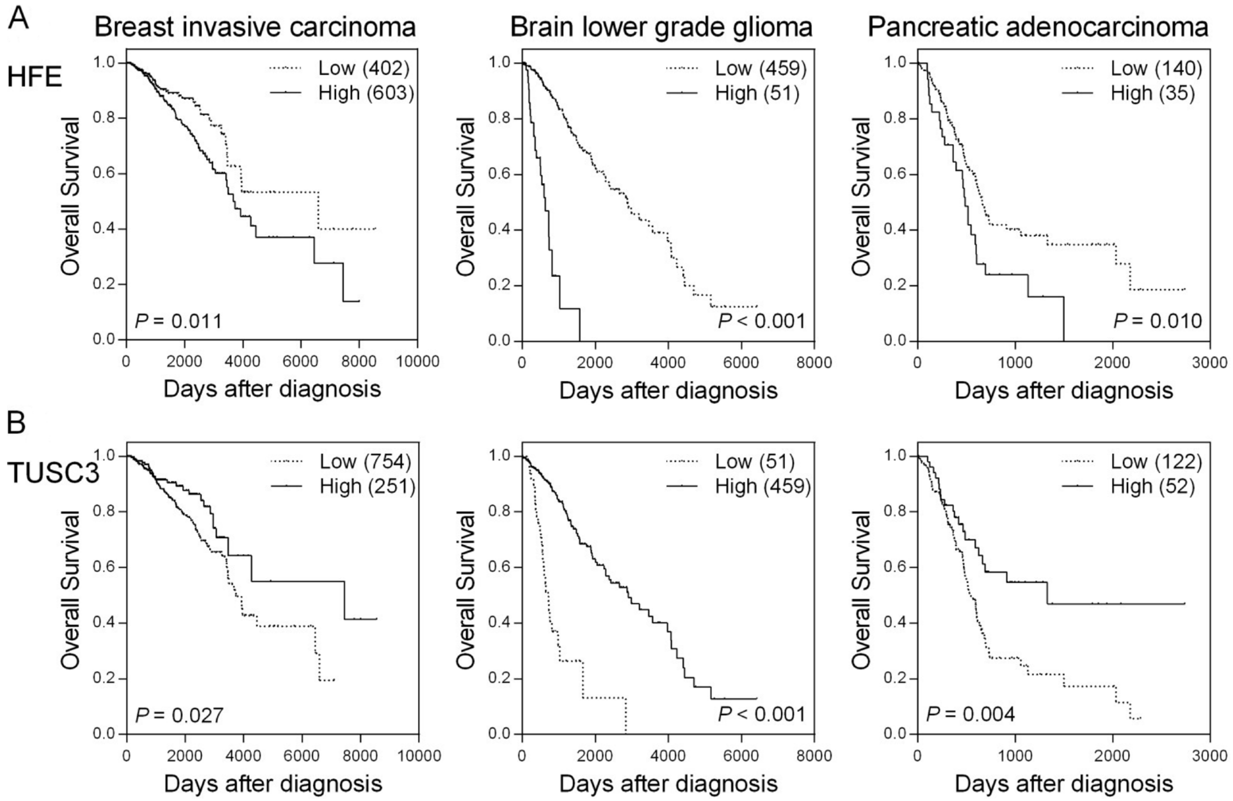

Supplementary Figure 2: Kaplan-Meier analysis of overall survival according to (**A**) *HFE* and (**B**) *TUSC3* expressions in TCGA breast invasive carcinoma, brain lower grade glioma, and pancreatic adenocarcinoma datasets.

Supplementary Table 1: Genotyped SNPs and the *P* values of their association with TTP in localized prostate cancer. See Supplementary_Table_1

Supplementary Table 2: Genotyped SNPs and the *P* values of their association with TTP in advanced prostate cancer. See Supplementary_Table_2
