# Supplementary material for: Vitamin D receptor-binding site variants affect prostate cancer progression
Source: Oncotarget. 2017 May 26;8(43):74119–28. doi: 10.18632/oncotarget.18271 (PMC5650327; doi:10.18632/oncotarget.18271)
Supplement: Supplementary file 2 [file oncotarget-08-74119-s002.pdf]

Supplementary Table 1: Genotyped SNPs and the *P* values of their association with TTP in localized prostate cancer

SNP ID	Chromosome	Position	Additive	Dominant	Recessive
rs10789077	chr1	59743939	0.115	0.302	-
rs17414204	chr1	81311994	0.726	0.927	0.050
**rs316052**	chr1	98724652	0.891	0.662	**0.015**
rs17668726	chr1	193646576	0.548	0.410	-
rs6673734	chr1	214758060	0.532	0.216	0.350
rs1490333	chr1	216274592	0.677	0.753	0.705
rs356992	chr2	60607097	0.598	0.523	0.937
rs950965	chr2	66521813	0.288	0.589	0.168
rs10198409	chr2	156464481	0.271	0.350	0.401
rs9863011	chr3	87069510	0.978	0.866	0.713
rs798580	chr3	119992658	0.568	0.598	0.679
rs1515372	chr3	124376943	0.223	0.380	0.208
rs12631628	chr3	159660117	0.698	0.843	0.495
rs10936548	chr3	169204342	0.984	0.757	0.353
rs11099242	chr4	134606625	0.503	0.495	0.737
rs10473995	chr5	77548993	0.803	0.899	0.717
rs6872228	chr5	124368580	0.492	0.553	0.600
rs10475856	chr5	166968159	0.921	0.825	0.849
**rs9393682**	chr6	26165029	**0.003**	**0.005**	**0.030**
rs10943438	chr6	78066555	0.608	0.415	0.877
rs2501639	chr6	92576658	0.282	0.544	0.209
**rs4499937**	chr6	98599730	0.552	0.266	**0.013**
rs17716922	chr6	103953259	0.535	0.594	0.570
rs1045530	chr7	32874660	0.747	0.885	0.639
rs3801232	chr7	42219838	0.122	0.112	0.640
rs9641549	chr7	115134888	0.879	0.757	0.947
**rs12671349**	chr7	132175098	**0.031**	**0.030**	-
**rs12532853**	chr7	146939689	0.123	**0.013**	0.107
rs1378033	chr8	14984517	0.722	0.430	0.372
rs13252746	chr8	78806369	0.632	0.800	0.209
rs997694	chr9	16474870	0.143	0.171	0.360
rs1930040	chr9	27928085	0.936	0.830	0.374
rs6559417	chr9	80662100	0.230	0.091	0.820
rs9329292	chr10	2537866	0.739	0.415	0.649
rs11256715	chr10	10712073	0.858	0.527	0.091
rs10995850	chr10	65683855	0.472	0.664	-
rs2394324	chr10	68512374	0.276	0.694	0.152
rs2574789	chr10	78521348	0.140	0.317	0.093

rs7074044	chr10	124842905	0.354	0.375	0.607
rs2387992	chr10	130651786	0.806	0.651	0.868
rs575050	chr11	85017190	0.118	0.167	0.222
rs2060756	chr12	40395375	0.931	0.736	0.631
rs1465057	chr12	51899148	0.807	0.666	0.906
rs7302357	chr12	52281805	0.735	0.865	0.597
rs2406254	chr12	85616797	0.199	0.264	0.307
rs9527770	chr13	57660493	0.913	0.810	0.369
rs17115183	chr14	29207158	0.572	0.729	-
rs7151113	chr14	33232896	0.511	0.226	0.226
rs213560	chr14	77707744	0.171	0.365	-
rs8032707	chr15	35572205	0.166	0.104	0.711
rs2291278	chr15	69902105	0.386	0.210	0.684
rs1834212	chr15	93876557	0.293	0.194	-
rs716820	chr17	47985581	0.425	0.425	0.651
rs7238440	chr18	21364727	0.457	0.662	0.344
**rs7226824**	chr18	25930825	0.648	0.266	**0.039**
rs2426477	chr20	51300670	0.676	0.609	0.891
rs7261371	chr20	57431233	0.380	0.380	-
rs11088247	chr21	33525119	0.204	0.624	0.083
rs6640615	chrX	10107276	0.652		
rs1060063	chrX	40350760	0.989		
rs3747440	chrX	133134250	0.649		
rs306885	chrX	154651506	0.931		

P values for log-rank test

-, not calculated because the frequency of the rare homozygote was below 0.05

$P < 0.05$ is in boldface
