# Supplementary material for: Vitamin D receptor-binding site variants affect prostate cancer progression
Source: Oncotarget. 2017 May 26;8(43):74119–28. doi: 10.18632/oncotarget.18271 (PMC5650327; doi:10.18632/oncotarget.18271)
Supplement: Supplementary file 3 [file oncotarget-08-74119-s003.pdf]

Supplementary Table 2: Genotyped SNPs and the *P* values of their association with TTP in advanced prostate cancer

SNP ID	Chromosome	Position	Additive	Dominant	Recessive
rs10789077	chr1	59743939	0.698	0.387	-
rs17414204	chr1	81311994	0.221	0.465	0.052
rs316052	chr1	98724652	0.569	0.997	0.133
rs17668726	chr1	193646576	0.170	0.231	-
rs6673734	chr1	214758060	0.576	0.680	0.580
rs1490333	chr1	216274592	0.087	0.358	0.050
rs356992	chr2	60607097	0.980	0.831	0.660
rs950965	chr2	66521813	0.660	0.822	0.581
rs10198409	chr2	156464481	0.783	0.403	0.303
rs9863011	chr3	87069510	0.326	0.169	0.974
rs798580	chr3	119992658	0.421	0.408	0.626
rs1515372	chr3	124376943	0.968	0.772	0.599
rs12631628	chr3	159660117	0.943	0.685	0.489
rs10936548	chr3	169204342	0.876	0.893	0.900
rs11099242	chr4	134606625	0.608	0.607	0.786
rs10473995	chr5	77548993	0.999	0.699	0.550
rs6872228	chr5	124368580	0.302	0.100	0.602
rs10475856	chr5	166968159	0.687	0.314	0.410
rs9393682	chr6	26165029	0.625	0.759	0.250
rs10943438	chr6	78066555	0.928	0.680	0.421
rs2501639	chr6	92576658	0.297	0.522	0.268
**rs4499937**	chr6	98599730	**0.029**	**0.022**	0.276
rs17716922	chr6	103953259	0.662	0.993	0.091
rs1045530	chr7	32874660	0.109	0.108	0.438
rs3801232	chr7	42219838	0.416	0.263	0.942
rs9641549	chr7	115134888	0.883	0.742	0.545
rs12671349	chr7	132175098	0.488	0.537	-
rs12532853	chr7	146939689	0.754	0.845	0.719
**rs1378033**	chr8	14984517	**0.032**	**0.018**	0.649
rs13252746	chr8	78806369	0.682	0.915	0.206
rs997694	chr9	16474870	0.975	0.785	0.576
rs1930040	chr9	27928085	0.979	0.660	0.113
rs6559417	chr9	80662100	0.983	0.992	0.961
rs9329292	chr10	2537866	0.458	0.600	0.467
**rs11256715**	chr10	10712073	0.388	0.774	**0.039**
rs10995850	chr10	65683855	0.769	0.791	-
rs2394324	chr10	68512374	0.808	0.862	0.824
rs2574789	chr10	78521348	0.206	0.389	0.176

rs7074044	chr10	124842905	0.326	0.210	0.935
rs2387992	chr10	130651786	0.677	0.737	0.728
rs575050	chr11	85017190	0.468	0.499	0.628
rs2060756	chr12	40395375	0.788	0.762	0.479
rs1465057	chr12	51899148	0.804	0.625	0.794
rs7302357	chr12	52281805	0.223	0.182	0.639
rs2406254	chr12	85616797	0.907	0.377	0.284
rs9527770	chr13	57660493	0.663	0.572	0.911
rs17115183	chr14	29207158	0.299	0.320	-
rs7151113	chr14	33232896	0.895	0.934	0.857
rs213560	chr14	77707744	0.751	0.430	-
rs8032707	chr15	35572205	0.176	0.065	0.874
rs2291278	chr15	69902105	0.533	0.502	0.812
**rs1834212**	chr15	93876557	0.063	**0.047**	-
rs716820	chr17	47985581	0.495	0.236	0.767
rs7238440	chr18	21364727	0.782	0.205	0.174
rs7226824	chr18	25930825	0.674	0.658	0.810
rs2426477	chr20	51300670	0.768	0.934	0.673
rs7261371	chr20	57431233	0.306	0.306	-
rs11088247	chr21	33525119	0.384	0.253	0.915
rs6640615	chrX	10107276	0.091		
rs1060063	chrX	40350760	0.966		
rs3747440	chrX	133134250	0.315		
rs306885	chrX	154651506	0.596		

P values for log-rank test

-, not calculated because the frequency of the rare homozygote was below 0.05

$P < 0.05$ is in boldface
